# National Trends in Statin Use by Coronary Heart Disease Risk Category

**DOI:** 10.1371/journal.pmed.0020123

**Published:** 2005-05-31

**Authors:** Jun Ma, Niraj L Sehgal, John Z Ayanian, Randall S Stafford

**Affiliations:** 1Stanford Prevention Research Center, Stanford University School of MedicineStanford, CaliforniaUnited States of America; **2**Division of General Medicine, Brigham and Women's Hospital and Department of Health Care PolicyHarvard Medical School, Boston, MassachusettsUnited States of America; University College DublinIreland

## Abstract

**Background:**

Only limited research tracks United States trends in the use of statins recorded during outpatient visits, particularly use by patients at moderate to high cardiovascular risk.

**Methods and Findings:**

Data collected between 1992 and 2002 in two federally administered surveys provided national estimates of statin use among ambulatory patients, stratified by coronary heart disease risk based on risk factor counting and clinical diagnoses. Statin use grew from 47% of all lipid-lowering medications in 1992 to 87% in 2002, with atorvastatin being the leading medication in 2002. Statin use by patients with hyperlipidemia, as recorded by the number of patient visits, increased significantly from 9% of patient visits in 1992 to 49% in 2000 but then declined to 36% in 2002. Absolute increases in the rate of statin use were greatest for high-risk patients, from 4% of patient visits in 1992 to 19% in 2002. Use among moderate-risk patients increased from 2% of patient visits in 1992 to 14% in 1999 but showed no continued growth subsequently. In 2002, 1 y after the release of the Adult Treatment Panel III recommendations, treatment gaps in statin use were detected for more than 50% of outpatient visits by moderate- and high-risk patients with reported hyperlipidemia. Lower statin use was independently associated with younger patient age, female gender, African American race (versus non-Hispanic white), and non-cardiologist care.

**Conclusion:**

Despite notable improvements in the past decade, clinical practice fails to institute recommended statin therapy during many ambulatory visits of patients at moderate-to-high cardiovascular risk. Innovative approaches are needed to promote appropriate, more aggressive statin use for eligible patients.

## Abstract 2

The prescribing of statins shows great variation among different types of patients, with many who would benefit receiving neither lifestyle counseling nor appropriate drugs.

## Introduction

Coronary heart disease (CHD) remains the leading cause of morbidity and mortality in the United States and is associated with substantial economic cost [1]. Hyperlipidemia represents an important modifiable risk factor in the development and progression of CHD. Estimates indicate that nearly 100 million American adults have total blood cholesterol levels of greater than 5.17 mmol/l (200 mg/dl) with 40% having levels greater than 6.21 mmol/l (240 mg/dl) [2]. Identification and treatment of patients with hyperlipidemia play an essential role in the primary and secondary prevention of CHD.

Currently, evidence-based practice guidelines focus on low-density lipoprotein cholesterol (LDL-C) as the primary target for risk reduction therapy and recommend that the intensity and target goals of LDL-C-lowering therapy should be adjusted to individual absolute risk for CHD [3]. Absolute CHD risk is categorized as low, moderate, or high based on the presence or absence of CHD, CHD-equivalent conditions, and major risk factors other than LDL-C. While therapeutic lifestyle changes are integral to general risk reduction, drug treatment proves necessary for selected patients whose absolute risk is high and/or whose LDL-C is inadequately controlled with lifestyle modifications alone. Among existing drug therapies, 3-hydroxy-3-methyl-glutaryl coenzyme A reductase inhibitors, more commonly known as statins, provide a generally well-tolerated and effective option for lowering LDL-C levels and decreasing the likelihood of subsequent CHD events [3,4].

Despite the compelling evidence of statins' therapeutic benefits, the literature abounds with documentation of wide treatment gaps in clinical practice [5–10]. Available research, however, offers only a limited understanding of how statin therapy varies by CHD risk, particularly for statin-eligible patients in the moderate-risk group. Also, national data are limited regarding recent changes in statin use.

Using serial cross-sectional data from 1992 through 2002, we tracked trends in statin use in the United States during ambulatory visits categorized by CHD risk, with or without a diagnosis of hyperlipidemia. In addition, we analyzed the independent associations of patient and physician characteristics with statin use for insights as to how to target interventions to improve statin use.

## Methods

### Data Sources

Annual data from1992 through 2002 were obtained from the National Ambulatory Medical Care Survey (NAMCS) and the outpatient department component of the National Hospital Ambulatory Medical Care Survey (NHAMCS). The National Center for Health Statistics provides complete descriptions of both surveys and yearly data at http://www.cdc.gov/nchs/about/major/ahcd/ahcd1.htm. These surveys, particularly NAMCS, have been validated against other data sources [11,12], and have also been utilized in past research of cholesterol management [13].

In brief, NAMCS captures health-care services provided by office-based physicians, while NHAMCS assesses services offered at hospital outpatient departments. Both surveys utilize multistage probability sampling procedures, enabling the generation of nationally representative estimates. Between 1992 and 2002, annual participation rates among physicians selected for NAMCS averaged 70%, while the participation rate in NHAMCS by selected hospitals with outpatient departments was 90%. In our study, we combined NAMCS and NHAMCS data to obtain a wider range of outpatient settings and a broader socioeconomic spectrum of patients seeking ambulatory care.

Standard encounter forms were completed for a systematic random sample of patient visits during randomly assigned reporting periods. Item nonresponse rates were mostly 5% or less in both surveys for all years. Yearly encounter forms varied slightly between NAMCS and NHAMCS and were revised every two years. Our analysis focused on domains of data that were consistently collected in both NAMCS and NHAMCS for the time period 1992–2002, including patient demographic and geography characteristics, reasons for visit (up to three), diagnoses (up to three), new and continuing medications (up to five in 1992–1994 and six in 1995–2002), and lifestyle counseling services provided or ordered at the visit.

### Participants

#### CHD risk categorization

We estimated CHD risk for adults aged 20 y and older based on risk factor counting. CHD risk was mutually exclusively categorized as low (0–1 risk factors), moderate (2+ risk factors), or high (CHD, other atherosclerotic diseases, or diabetes). The moderate-risk group included visits by patients without CHD or equivalent but with at least two of the following risk factors: age (for men, >45 y; for women, >55 y), cigarette smoking, or a physician-reported diagnosis of hypertension. Unfortunately, the other two major CHD risk factors—high-density lipoprotein cholesterol levels and family history of premature CHD—were not captured in either data source. Also, neither data source provided actual cholesterol measurements. Disease conditions were identified by International Classification of Disease (ICD-9) codes, as well as by the appropriate reason-for-visit codes that are specific to NAMCS and NHAMCS. For instance, we identified patients as having hyperlipidemia if their encounter forms contained an International Classification of Disease code within 272.0–272.4. For the sake of this study, patients whose encounter forms did not indicate the presence of a condition were assumed to not have that condition.

#### Patient visit characteristics.

Nonclincal characteristics included patient age, gender, race/ethnicity, medical insurance, visit status, United States census region, metropolitan area status, physician specialty, and practice setting. Medical insurance was classified as private/commercial, public (i.e., Medicare and Medicaid), or other (e.g., workers' compensation or self-pay). Visit status distinguished first-time visits from return visits to a practice. Physician specialty was available only from NAMCS, which contributed more than 90% of the total visits for each of the study years. We categorized physician specialties as cardiology, internal medicine, general and family practice, or other.

#### Measures

Of primary interest were the rate of statin use relative to CHD risk and the relationship of statin use to patient visit characteristics. The rate of statin use was calculated as the proportion of patient visits where a statin was reported (i.e., atorvastatin, lovastatin, pravastatin, simvastatin, or fluvastatin). Before its removal from the market in 2001, cerivastatin was used scarcely (<2% among visits by patients with hyperlipidemia) and therefore is not reported in this study. Measuring the rate of statin use by CHD risk category provided a relative indicator of appropriate prescribing patterns, that is, the prevalence of statin use should be highest among high-risk patients, for whom secondary prevention is a priority. Variations of statin use by patient visit characteristics, if detected, would reflect a lack of equity in processes of care in that uniform practices are expected unless evidence-based guidelines recommend otherwise.

#### Analyses

Statistical analyses were performed using SAS for Windows software (SAS Institute, Cary, North Carolina, United States) and SAS-callable SUDAAN software (RTI, Research Triangle Park, North Carolina, United States) to account for sampling weights and the complex survey design. The unit of analysis is the patient visit. We report national annual means of the rate of statin use by CHD risk category and corresponding 99% confidence intervals for the years 1992 through 2002. χ^2^ tests examined the association of statin use with individual patient visit characteristics for combined 1995–2002 NAMCS and NHAMCS data. The independent effect of each patient visit characteristic on statin use after controlling for all other characteristics was assessed with multivariate logistic regression.

## Results

In 2002, visits by patients at moderate or high risk involved higher proportions of older patients (mean age 65 y) than low-risk patient visits (mean age 51 y), and consequently were more likely to be covered by public insurance, particularly Medicaid (Table 1). Moderate- and high-risk patient visits also were made up of more men and return patients. In addition, a greater percentage of high-risk patient visits (11%) were seen by cardiologists than patient visits at low and moderate risk (2% and 4%, respectively). Internists and general and family practitioners played a dominant role in the care of moderate- and high-risk patients, accounting for 69% of visits by moderate-risk patients and 58% of visits by high-risk patients. Distributions by race/ethnicity, geographic region, residence area, and practice setting did not differ by CHD risk. Overall, the majority of patient visits were return visits to office-based physicians made by non-Hispanic whites and residents living within metropolitan statistic areas. Patient visits were distributed similarly across the four geographic regions, with a slightly higher proportion from the southern region.

**Table 1:**
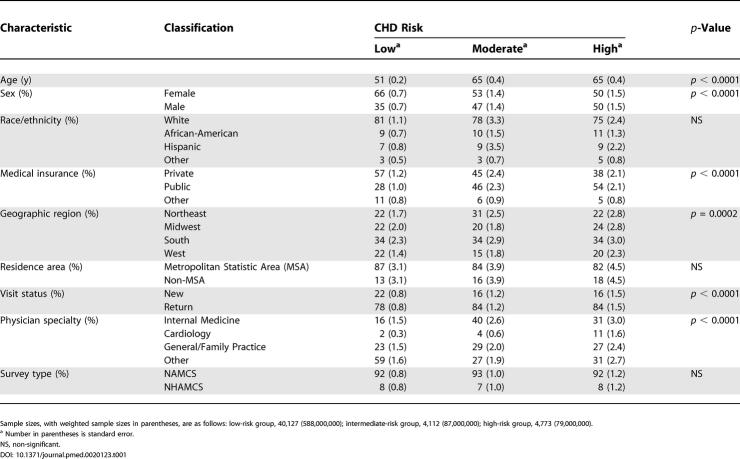
Differences in Patient, Physician, and Visit Characteristics by CHD Risk in 2002

Sample sizes, with weighted sample sizes in parentheses, are as follows: low-risk group, 40,127 (588,000,000); intermediate-risk group, 4,112 (87,000,000); high-risk group, 4,773 (79,000,000).

^a^ Number in parentheses is standard error.

NS, non-significant.

**Figure 1:**
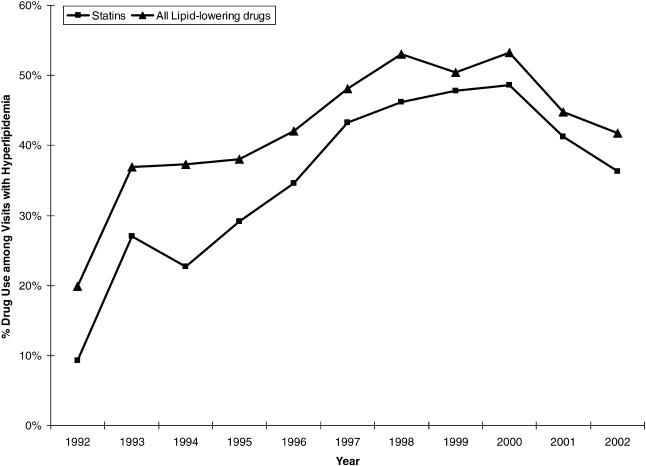
Use of Statins and All Lipid-Lowering Medications among United States Ambulatory Visits by Patients Diagnosed with Hyperlipidemia Data from NAMCS and NHAMCS, 1992–2002.

**Figure 2:**
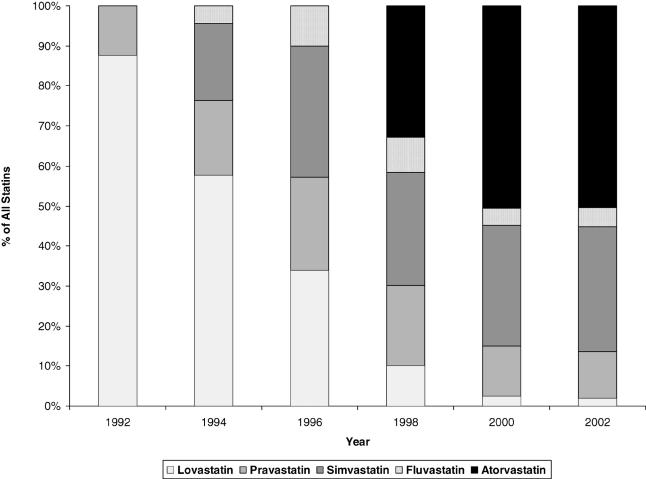
Share of Total Statin Use among United States Ambulatory Visits by Individual Statin Medications Data from NAMCS and NHAMCS, 1992–2002.

Throughout the study period, statins were primarily used among patients whose visit involved reported hyperlipidemia, representing 97% of all statin use in 1992 and 91% in 2002. Statin use increased nearly 5-fold from 9% (99% confidence interval: 7%–12%) of all visits with reported hyperlipidemia in 1992 to 49% (42%–55%) in 2000, but then declined to 36% (31%–42%) in 2002 (Figure 1). Of note, however, the annual rate of increase in frequency of patient visits with reported hyperlipidemia was 34% in 2001 and 21% in 2002, while it averaged only 12% through 2000. The dominance of statins as lipid-lowering agents grew markedly from 47% of all lipid-lowering medications in 1992 to 87% in 2002 (Figure 1). Among available statins, lovastatin remained the therapeutic choice through 1996, after which it was surpassed by other statins, particularly simvastatin and then atorvastatin (Figure 2). Atorvastatin constituted 51% (46%–56%) and simvastatin 32% (27%–36%) of all statin use in 2002.

**Figure 3:**
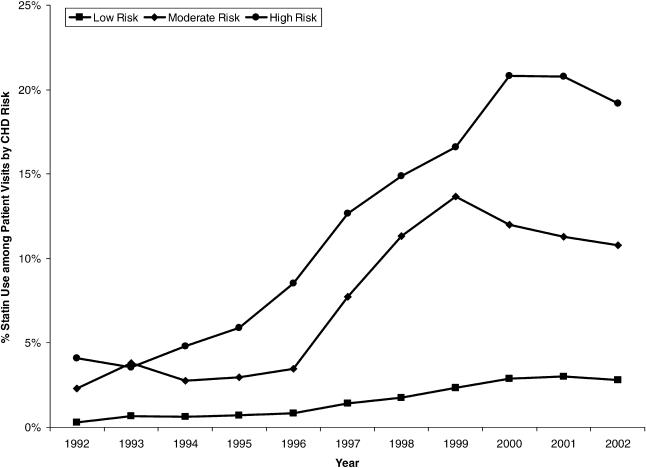
Use of Statins among United States Ambulatory Visits, by CHD Risk Category Data from NAMCS and NHAMCS, 1992–2002.

As expected, high CHD risk patient visits resulted in greater statin use, and the divergence in statin use among the three risk categories has grown in recent years. Absolute increases in the rate of statin use were greatest for high-risk patient visits with or without reported hyperlipidemia—a 15 percentage-point increase from 4% of all visits in 1992 to 19% in 2002—followed by a nine percentage-point increase (2% to 11%) for moderate-risk patient visits and a 2.5 percentage-point increase (0.3% to 2.8%) for low-risk patient visits (Figure 3). Statin use in the moderate-risk group peaked at 14% (10%–17%) in 1999. Similarly, the rate of statin use in the high-risk group declined slightly from 2001 to 2002.

Among patient visits with reported hyperlipidemia, statins were used in 14% (8%–19%) of high-risk visits and 9% (5%–14%) of moderate-risk visits in 1992. The high-risk group's statin use rate rose to 60% (49%–71%) in 2000 and was 50% (40%–61%) in 2002. Likewise, the rate in the moderate-risk group climbed to 56% (42%–70%) in 1999 and stabilized at 44% (32%–57%) in 2002. In addition, lifestyle counseling (i.e., regarding diet, exercise, or smoking cessation) occurred in only 43% (32%–53%) of new and general medical examination visits in 2002 for patients who had moderate CHD risk and were diagnosed with hyperlipidemia. Improvements over time in counseling rates were minimal.

The increase in statin use with CHD risk and with the year of study persisted after controlling for physician-reported hyperlipidemia, number of medications, and nonclinical patient visit characteristics (Table 2). Moderate- to high-risk patient visits had a 1.2- to 2.5-fold greater likelihood of taking a statin relative to visits by patients at low risk. Statin use was approximately three times as likely in 2001 and 2002 as in 1995 and 1996. Additionally, lower statin use was independently associated with younger patient age, female gender, African American background (versus non-Hispanic white), non-cardiologist care, and fewer total reported medications.

**Table 2:**
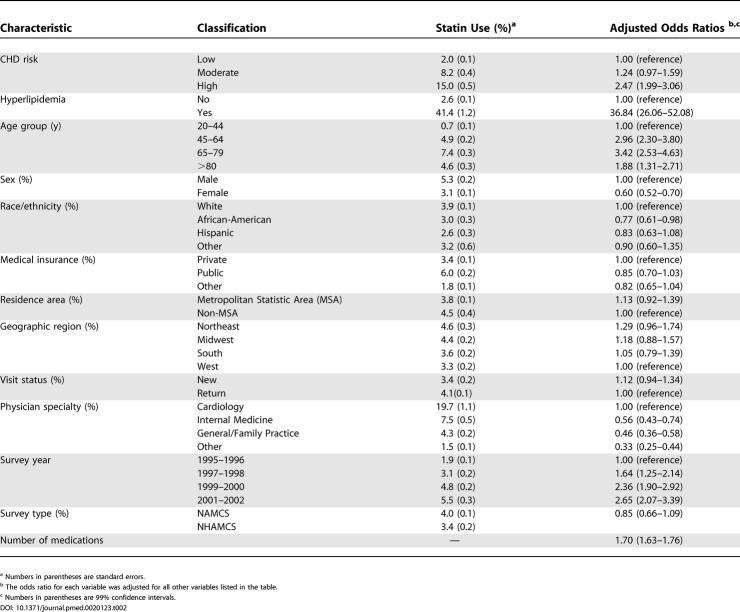
Predictors of Statin Use with Combined 1995–2002 NAMCS and NHAMCS Data

^a^ Numbers in parentheses are standard errors.

^b^ The odds ratio for each variable was adjusted for all other variables listed in the table.

^c^ Numbers in parentheses are 99% confidence intervals.

## Discussion

Despite significant increases from 1992 to 2002 in use of statins associated with hyperlipidemic patient visits, the magnitude of increases is smaller than expected and the rate of use remains suboptimal according to the best available evidence. The underuse of statins is most prominent among visits by patients at high or moderate risk of CHD who do not have a physician-noted diagnosis of hyperlipidemia but may nonetheless be eligible for lipid-lowering drug therapy. Previous research reports that physicians are more likely to diagnose hyperlipidemia if laboratory reports show abnormal lipid levels [14]. However, the normal ranges of lipid levels on many laboratory reports do not take into account individual patients' absolute risk.

When evaluating statin use across different CHD risk categories, the observed trends raise several issues. Both the rate of statin use and the absolute increases in the rate over time were positively associated with the level of CHD risk, which appropriately conforms to the notion of risk stratification. The associations persisted after adjusting for potentially confounding factors such as a hyperlipidemia diagnosis and nonclinical patient visit characteristics. Even so, in 2002, one year after the publication of Adult Treatment Panel III [3], statins were reportedly used in only 19% of patient visits with established CHD or its equivalents, and the average rate was no higher than 50% among high-risk visits where a diagnosis of hyperlipidemia also was noted. These data suggest a dramatic treatment gap. Another analysis based on national data estimated that 72% of Americans with existing CHD would benefit from drug therapy to achieve the target LDL-C goal of 2.59 mmol/l (100 mg/dl) or less, assuming a 10% LDL-C reduction with diet [15]. However, only 11% of those eligible individuals received lipid-lowering drug therapy, suggesting a gap of 89% [15]. These obvious treatment gaps are disconcerting, especially in light of the recent Adult Treatment Panel III update[16] that supports more intensive lipid-lowering drug therapy for patients at high and moderately high risk for a heart attack. Barriers to adequate treatment of high-risk patients may stem from the patient (e.g., lack of drug adherence, concern about adverse effects, inadequate knowledge of their hyperlipidemia, and drug cost), the physician (e.g., lack of guideline awareness, failure to measure lipid levels, and overestimation of actual treatment), and the health-care system (e.g., lack of monitoring and follow-up and emphasis on acute medical problems) [10]. If the current practice continues, the observed treatment gaps are expected to persist or even widen.

While statins deliver the greatest benefits when used for secondary prevention, evidence continues to accumulate that suggests an important role of statins in the primary prevention of cardiovascular events, particularly for patients at increased risk [6,17]. Our data show an increase in statin use from 2% of moderate-risk patient visits in 1992 to 14% in 1999, but without continued growth subsequently. Optimal proportions could not be determined because of the lack of detailed clinical data. Nonetheless, National Health and Nutrition Examination Survey III data showed that 60% of 38.5 million adult Americans without CHD who had two or more risk factors had an LDL-C level above the recommended 3.36 mmol/l (130 mg/dl) and that 45% would remain eligible for drug therapy even after a 10% decrease in LDL-C with diet [15]. In addition, Fedder and colleagues found a doubling effect in the number eligible for primary prevention drug therapy by switching to Framingham risk scoring [18]. Other researchers have reported that the proportions of treatment-eligible primary prevention patients who received no drug therapy reached as high as 97% [5,19]. In our study, statin use was reported in only 44% of moderate-risk patient visits for which a diagnosis of hyperlipidemia was noted, which is surprisingly low given that the entire group would be expected to benefit from statin therapy. We also concur with other researchers who have discussed the role that inadequate lifestyle counseling plays in the existing cholesterol treatment gaps [10,13]. Our data show that lifestyle counseling occurred during fewer than 50% of new and general medical examination visits by moderate-risk patients, even though these types of visits arguably represent better opportunities for counseling services than return, illness-focused visits.

It is intriguing to note that earlier increases in statin use were not sustained in 2001 and 2002. Studies using alternative data sources are needed to corroborate this observation, and detailed market research is necessary for understanding the underlying causes of this unexpected decline in use. We speculate that the observed trends may be partially explained by discordant rates of increase in the diagnosis of hyperlipidemia versus the prescribing of statins. Also, NAMCS and NHAMCS data released after 2002 will help determine whether the noted declines are due to random fluctuations in data reporting.

Wide gaps between evidence-based lipid-lowering therapy and physician practice were reported in many other western countries as well. For instance, a survey conducted in nine European countries found that only 32% of patients with confirmed CHD received lipid-lowering medications [20]. Likewise, in a population-based study from the Netherlands, merely 16% of individuals eligible for lipid-lowering drugs were actually treated [7].

In spite of being clearly underused, statins increasingly dominate lipid-lowering drug therapy, accounting for 92% of all lipid-lowering medications used in 2002, which confirms the trends seen in United States retail pharmacy dispensing data [2]. Also, in concert with other researchers [21], we observed a shift in the leading statin prescribed over time, from lovastatin to simvastatin and then to atorvastatin, corresponding to their market entry. Atorvastatin accounted for over half of all statin use in 2002. Even though most statins share similar tolerability, some evidence shows that atorvastatin has greater dose-specific potency for lowering LDL-C and total cholesterol [22].

Additionally, our data add support to available literature documenting inequities in use of statins for patients with different social and clinical characteristics [23–26]. Of particular note are the lower rates of statin use in at-risk younger patients, females, African-Americans, and patients cared for by non-cardiologists. These findings may be useful for guiding targeted interventions that aim to bring physician practice into agreement with published guidelines for cardiovascular risk reduction.

Our findings must be interpreted in the context of data limitations. Although both NAMCS and NHAMCS are designed to produce nationally representative estimates, these estimates are not linked to individuals but to patient visits. As a result, reported statin use may overestimate the actual administration because patients prescribed drug therapy likely make more visits because of greater disease severity and/or the need of frequent follow-ups. Also, we are missing people with risk factors who have not been seen by a physician or whose risk factors failed to be recorded. On the other hand, underestimation is also possible, for example, because of physicians' lack of awareness or incomplete reporting of patient medication uses. However, the failure to inquire or report an important agent such as a statin may be a clinical oversight in itself and contribute to therapeutic gaps. The degree of inaccuracy in our estimates is perhaps small, however, as suggested by the comparability of the current results to previous reports.

Lack of detailed clinical data prohibits accurate risk assessment based on Framingham risk scoring. While the risk factor counting algorithm that we used may simulate practical risk estimation by many physicians, it precludes the assessment of appropriateness of statin use in relation to the latest lipid-lowering guidelines. This creates difficulty interpreting the rate of statin use observed for the moderate-risk group. In particular, adequate information is not available to differentiate varying levels of absolute risk among the moderate-risk group. We likely misclassified some patients as moderate risk when they may have actually been high risk despite the absence of CHD or CHD equivalents. On the other hand, indications for statins might be marginal for some young patients with modestly elevated risk factors. A final caveat is that neither NAMCS nor NHAMCS captures patient compliance or outcomes, although these are perhaps separate issues from physician adherence to evidence-based medicine.

Despite the acknowledged limitations, NAMCS and NHAMCS cover a longer consecutive time span and provide more complete information about disease-specific physician activities than many other national data bases, e.g., the Medical Expenditures Panel Survey, the National Health Assessment Nutrition Examination Survey, and the National Health Interview Survey.

In conclusion, persistent gaps in statin therapy suggest a continued need for improved CHD risk stratification of all patients, and treatment with statins when indicated. Information technology and broader national policy around quality measurement and reporting are just two potential strategies that could be used to improve current practice. Patient-centered interventions should strengthen patient education and improve patient access to different treatment options. Interventions should be targeted to at-risk patients whose drug regimens need to be reassessed and to physicians, particularly non-cardiologists, whose practices need be improved. Guidelines for cardiovascular risk reduction treatment and determination of the specific patients who can benefit from statin therapy will continue to evolve. Indications for use in primary CHD prevention are likely to expand for statins. Given the observed practice shortfalls, drug therapy in moderate-risk patients remains an important priority for improvement.

**Patient Summary**

**Background**

Statins, a group of drugs that block a part of the pathway essential in forming lipids, have been shown to be very effective in reducing the chances of heart attacks and stroke both in people who have already had such an illness, and also in otherwise healthy people who have multiple risk factors. Long-term data are limited regarding whether people who might most benefit from such drugs are in fact receiving them.

**What Did the Investigators Do?**

Using two large United States outpatient care surveys that have run for many years, they looked at information on how doctors prescribed these drugs to patients. They found that between 1992 and 2002 the proportion of visits by patients with high lipid levels during which statins were prescribed rose from 9% in 1992 to 49% in 2000 and then fell to 36%. By 2002, statins were the dominant drug used for people with high lipid levels, accounting for 87% of all prescribed lipid-lowering medications. Although people at highest risk of cardiovascular diseases (with or without high lipid levels) were most likely to be prescribed a statin, by 2002, statins were prescribed in only 19% of visits by these people. In addition, only 11% of visits by people with multiple risk factors received a statin. Less than half the patient visits that arguably represent optimal opportunities for counseling services received counseling about how they might improve their cardiovascular health through lifestyle changes. Other factors that led to lower prescribing of statins were younger patient age, female gender, African American background, and care by non-cardiologists.

**What Do These Findings Mean?**

It seems that many people who could benefit from statins are not receiving them. Equally importantly, many people also did not receive information about how they could modify their lifestyle.

**Where Can I Get More Information?**

MedlinePlus has information on high lipids: go to http://www.nlm.nih.gov/medlineplus and search for “hyperlipidemia”

The British Heart Foundation has a number of pages on lipids: go to http://www.bhf.org.uk and search for “lipids”

## Abbreviations

CHD: coronary heart disease
LDL-C: low-density lipoprotein cholesterol
NAMCS: National Ambulatory Medical Care Survey
NHAMCS: National Hospital Ambulatory Medical Care Survey
